# Supplementary figures and images for: A deep generative model of 3D single-cell organization
Source: PLoS Comput Biol. 2022 Jan 18;18(1):e1009155. doi: 10.1371/journal.pcbi.1009155 (PMC8797242; doi:10.1371/journal.pcbi.1009155)

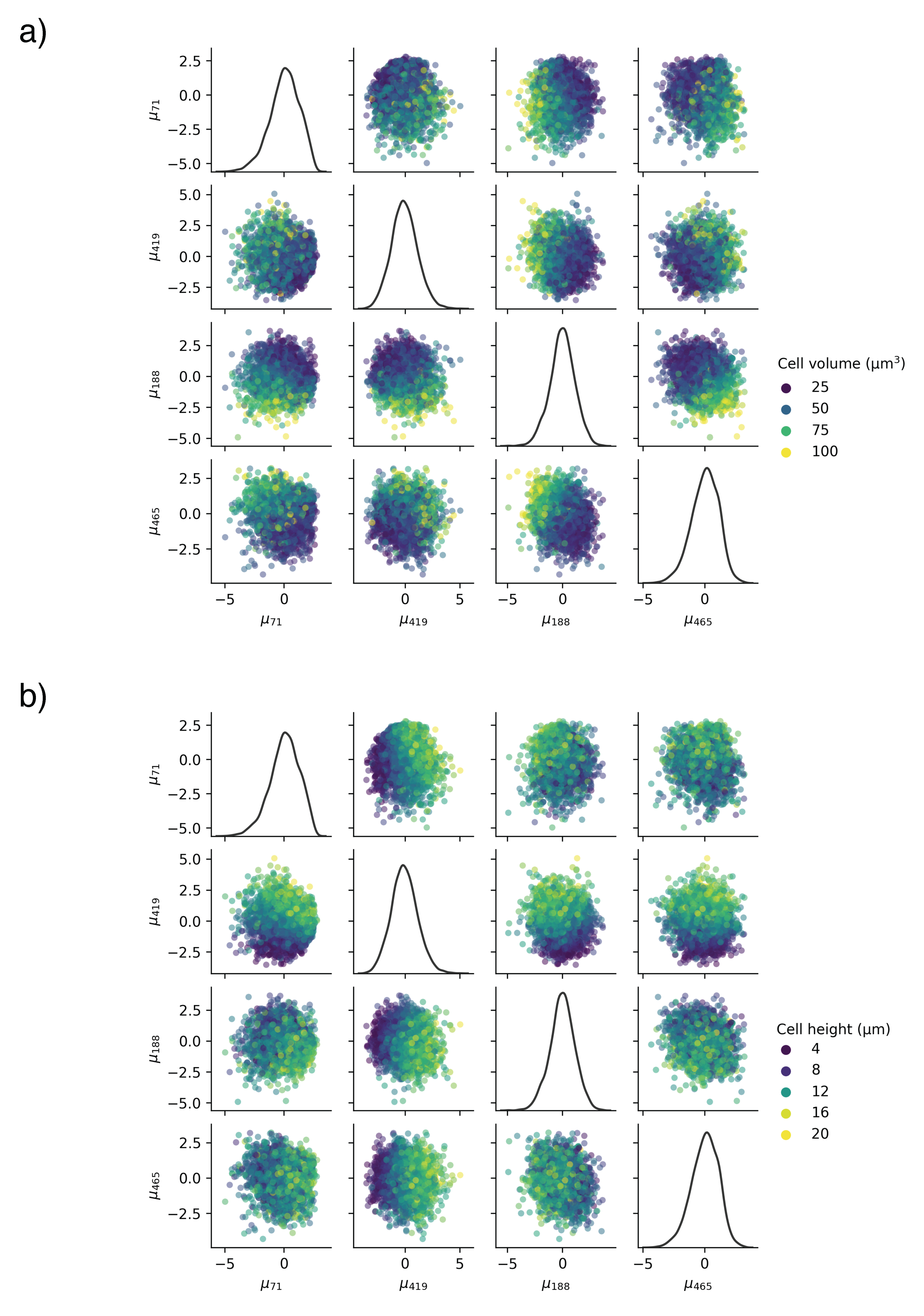

Supplement: S1 Fig — The marginal distribution of each latent dimension is plotted on the diagonal. a) Here we color by the cell volume, and see a visually apparent pattern in the data—most notably a strong correlation with μ188. b) Here we color by the cell height, and again observe structure in the scatter plots—most notably a strong correlation with μ419. (TIF) [file pcbi.1009155.s001.tif]

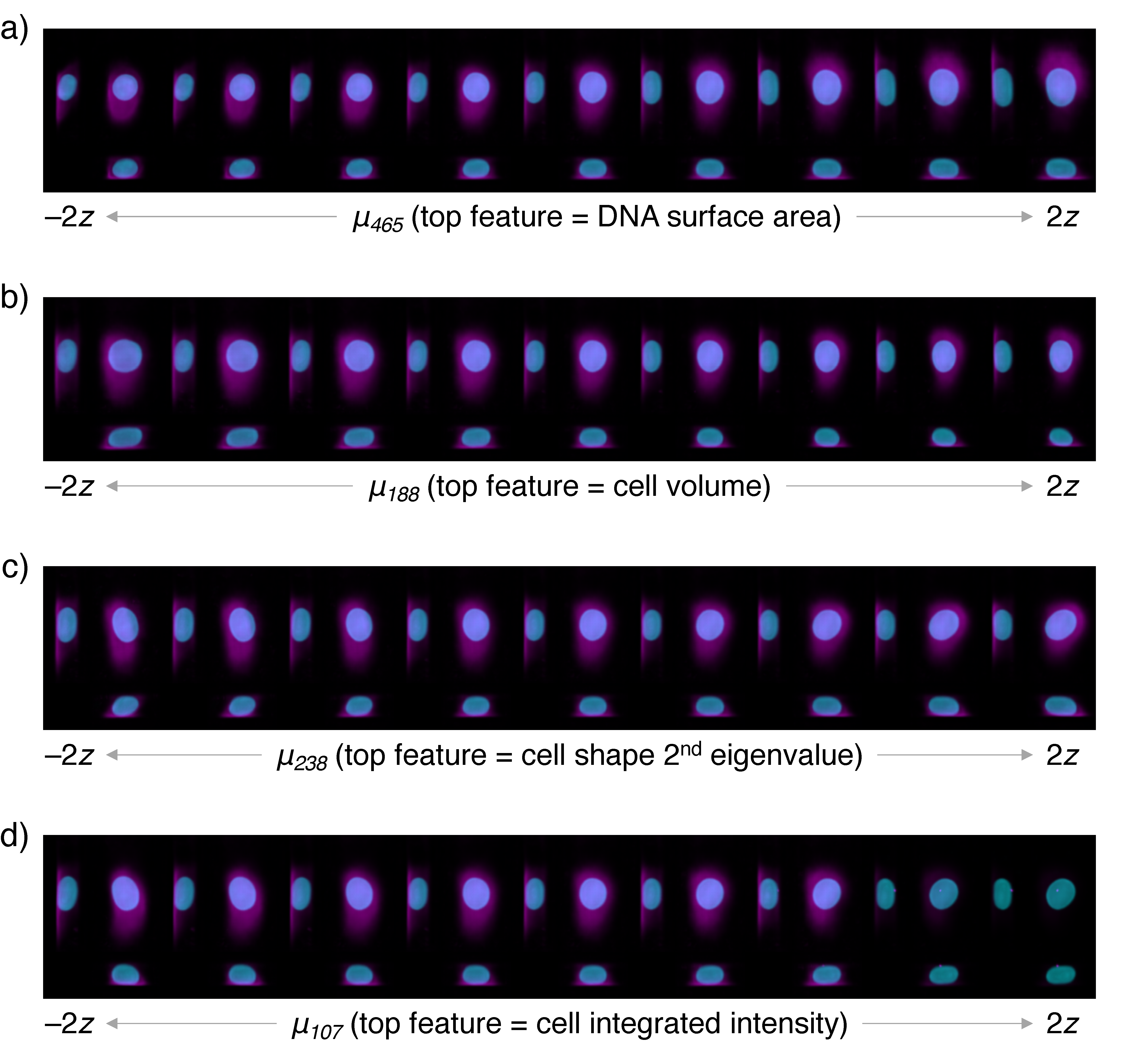

Supplement: S2 Fig — Walks are performed along the specified dimension in nine steps, starting at negative two standard deviations and ending at two standard deviations. All other latent dimensions are set to 0. We include the name of the most highly correlated cell feature, but the cell features are highly correlated (see S3 Fig) and a single latent space dimension may correlate with many cell features. a) Latent dimension μ465, which is most strongly correlated with nuclear surface area. b) Latent dimension μ188, which is most strongly correlated with cell volume. c) Latent dimension μ238, which is most strongly correlated with tilt/shear along the x-z-axes. d) Latent dimension μ107, which is most strongly correlated with the total integrated intensity in the plasma membrane dye channel. (TIF) [file pcbi.1009155.s002.tif]

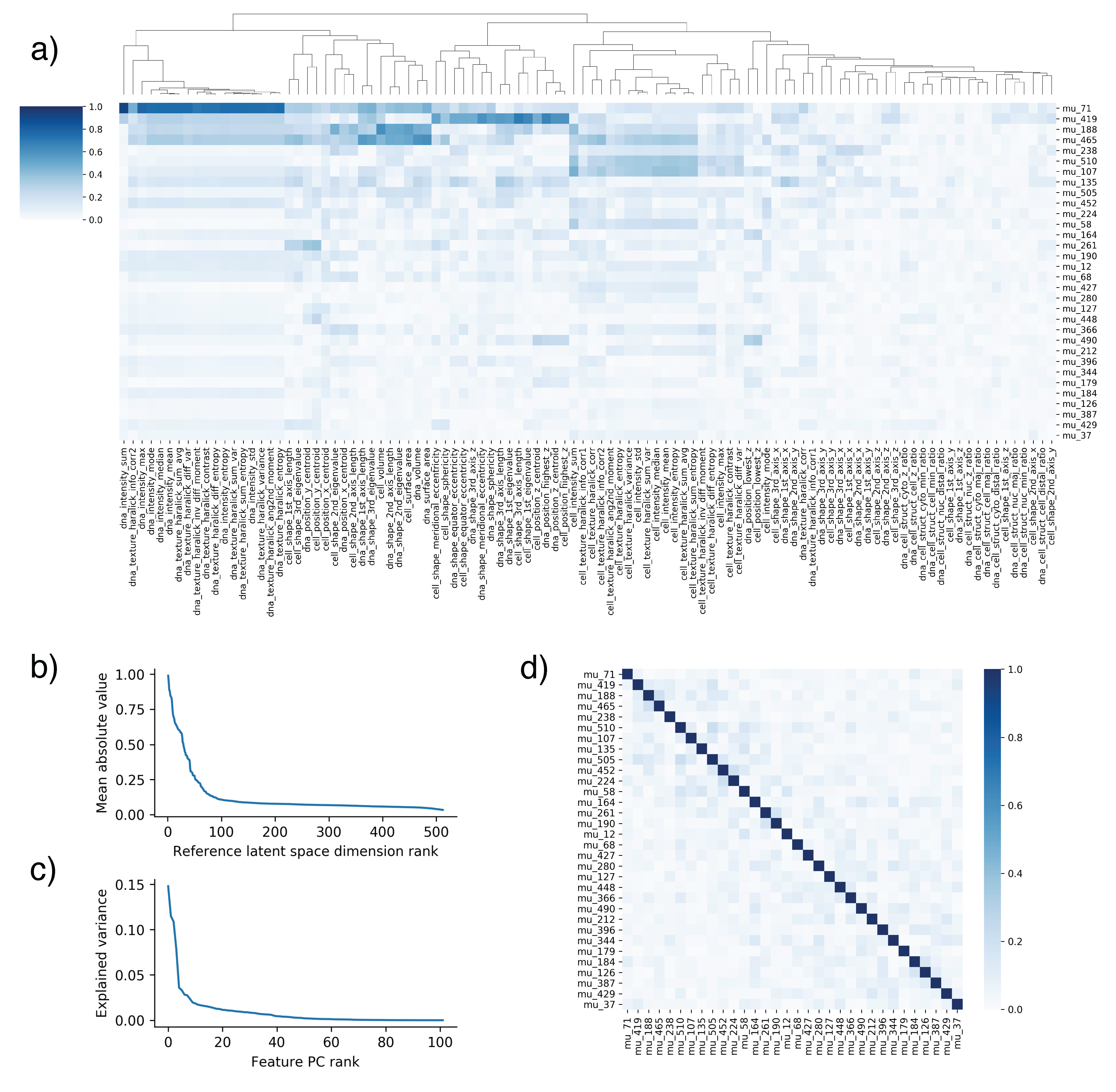

Supplement: S3 Fig — a) Heatmap of Spearman correlations of reference latent space dimensions with single-cell features on all cells in the test set. Cell features are hierarchically clustered. Latent space dimensions are sorted in descending rank by mean absolute deviation from 0, and for clarity only the top 32 dimensions are shown. Dimensions below 32 displayed significantly more noise and less correlation with cell features. b) Mean absolute deviation from 0 of all reference latent space dimensions, sorted by value. Values are computed by averaging over all cells in the test set. c) Explained variance of principal components of the z-scored cell features on all cells in the test set. d) Pearson correlation of the top 32 dimensions of the latent space, computed on all cells in the test set as ranked by mean absolute deviation from 0. We note that these dimensions display little to no correlation structure, empirically verifying the ability of the β-VAE to produce a disentangled latent space. (TIF) [file pcbi.1009155.s003.tif]

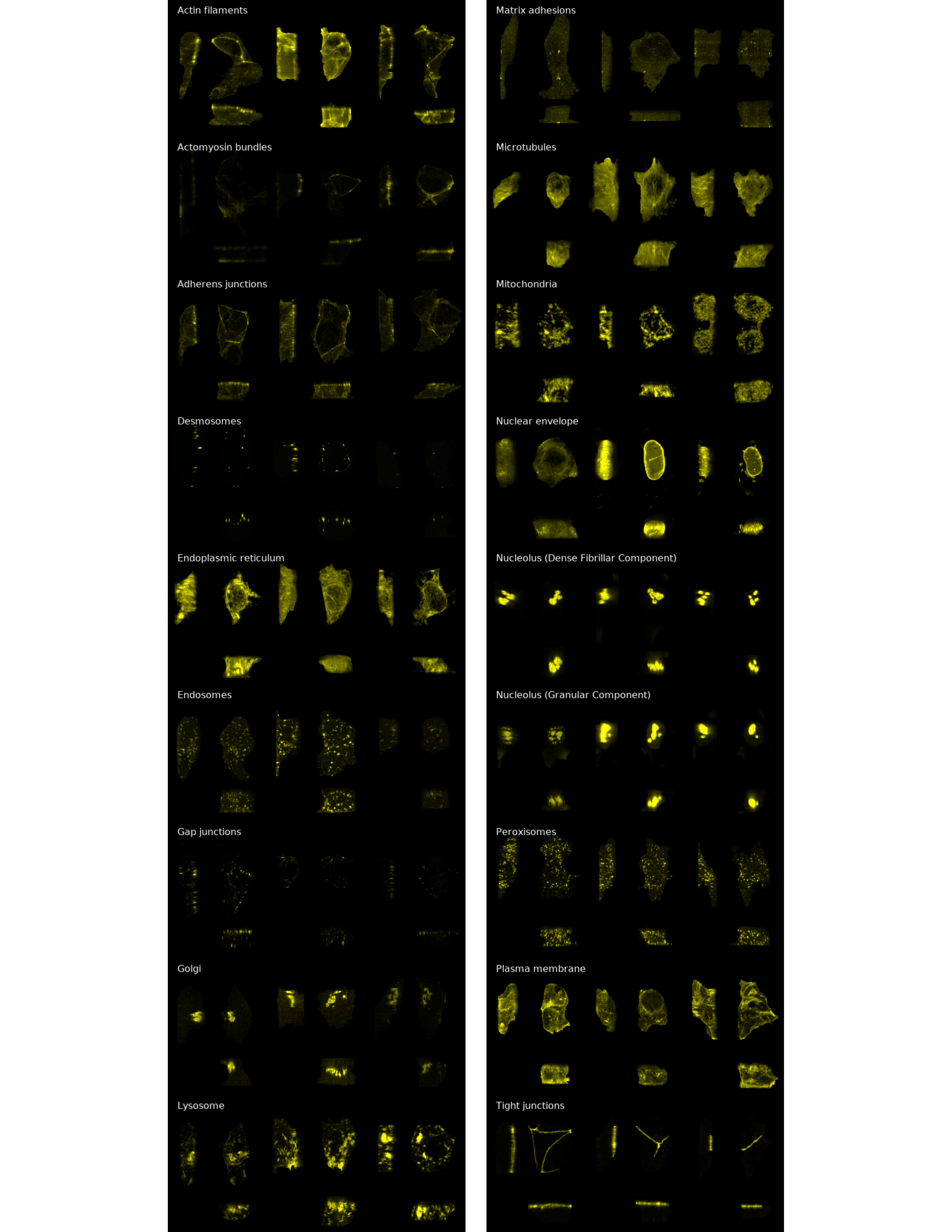

Supplement: S4 Fig — Each cell only has one mEGFP-tagged structure, so examples are all from different cells. (TIF) [file pcbi.1009155.s004.tif]

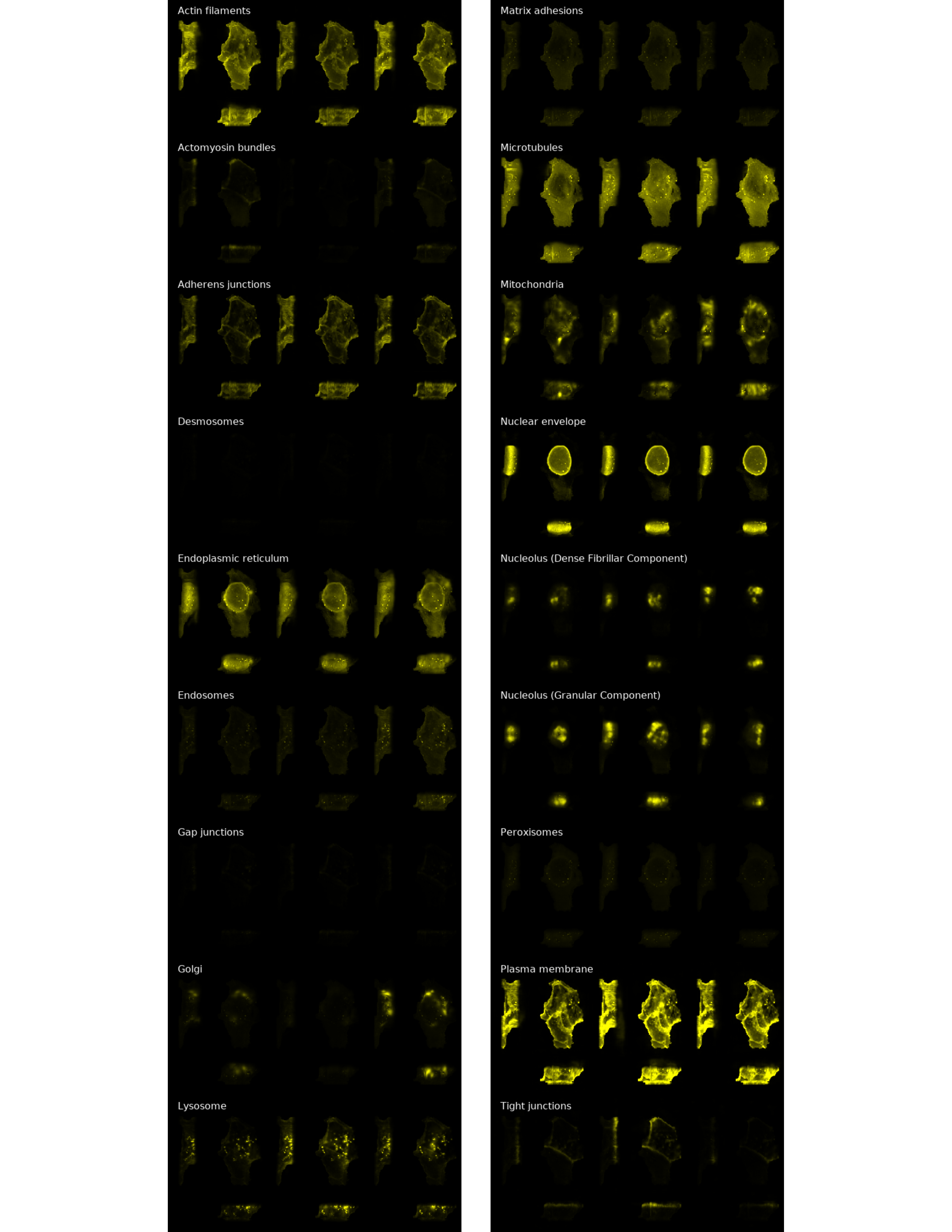

Supplement: S5 Fig — Three examples of each mEGFP-tagged structure are shown. Structures are generated using random draws from the conditional latent space, while keeping the reference geometry fixed to a single (randomly chosen) cell geometry from the test set. The same cell geometry is used across all structures shown here. (TIF) [file pcbi.1009155.s005.tif]

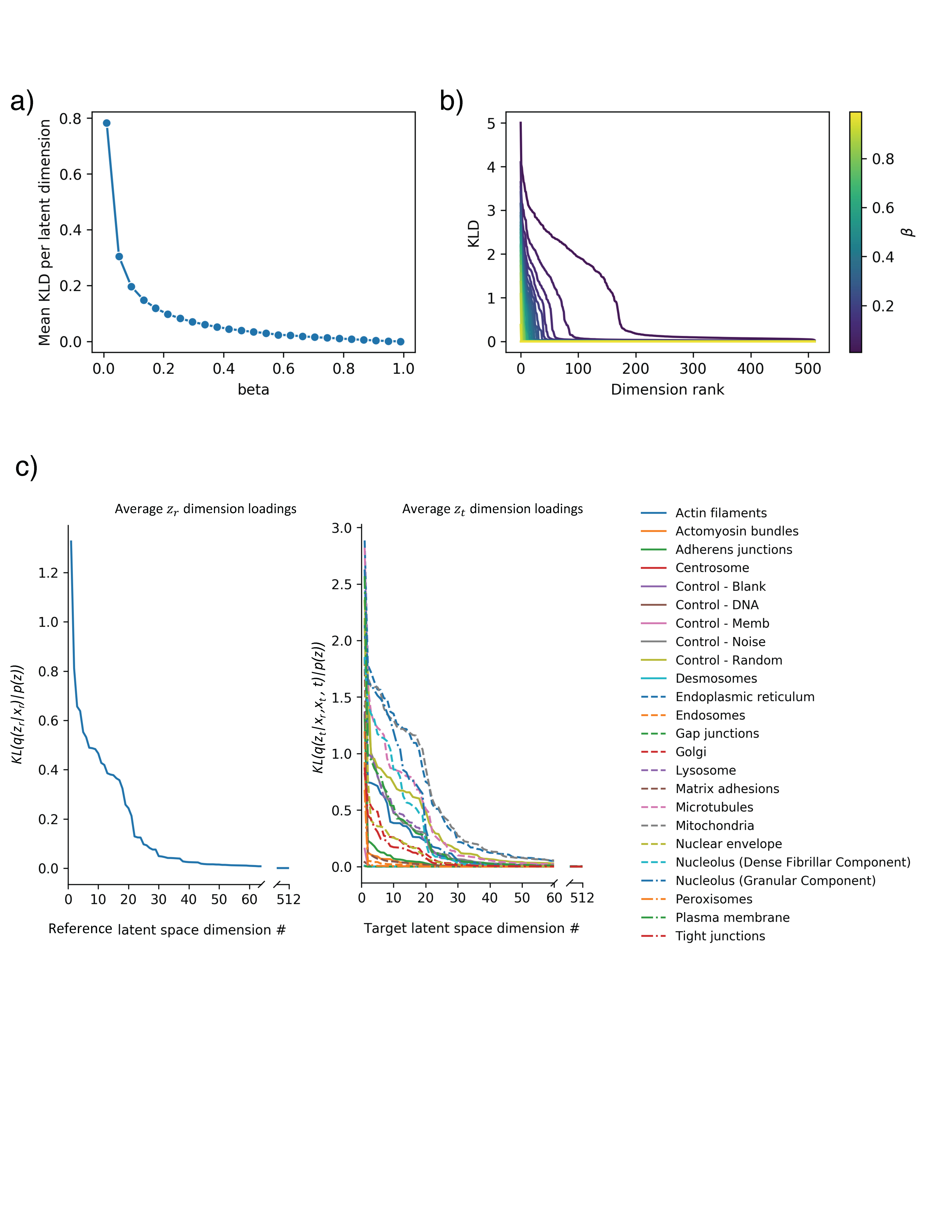

Supplement: S6 Fig — a) Mean KLD per dimension for the reference latent space of the test set in the 2D model, as a function of β, averaged over all dimensions in the latent space. b) Mean KLD per dimension for the reference latent space of the test set in the 2D model, as a function of dimension rank, for each model fit using a different β. c) Left: Mean KLD per dimension for the reference latent space of the test set in the 3D model, as a function of dimension rank. Right: Mean KLD per dimension for the conditional latent space of the test set in the 3D model, as a function of dimension rank and structure type. (TIF) [file pcbi.1009155.s006.tif]

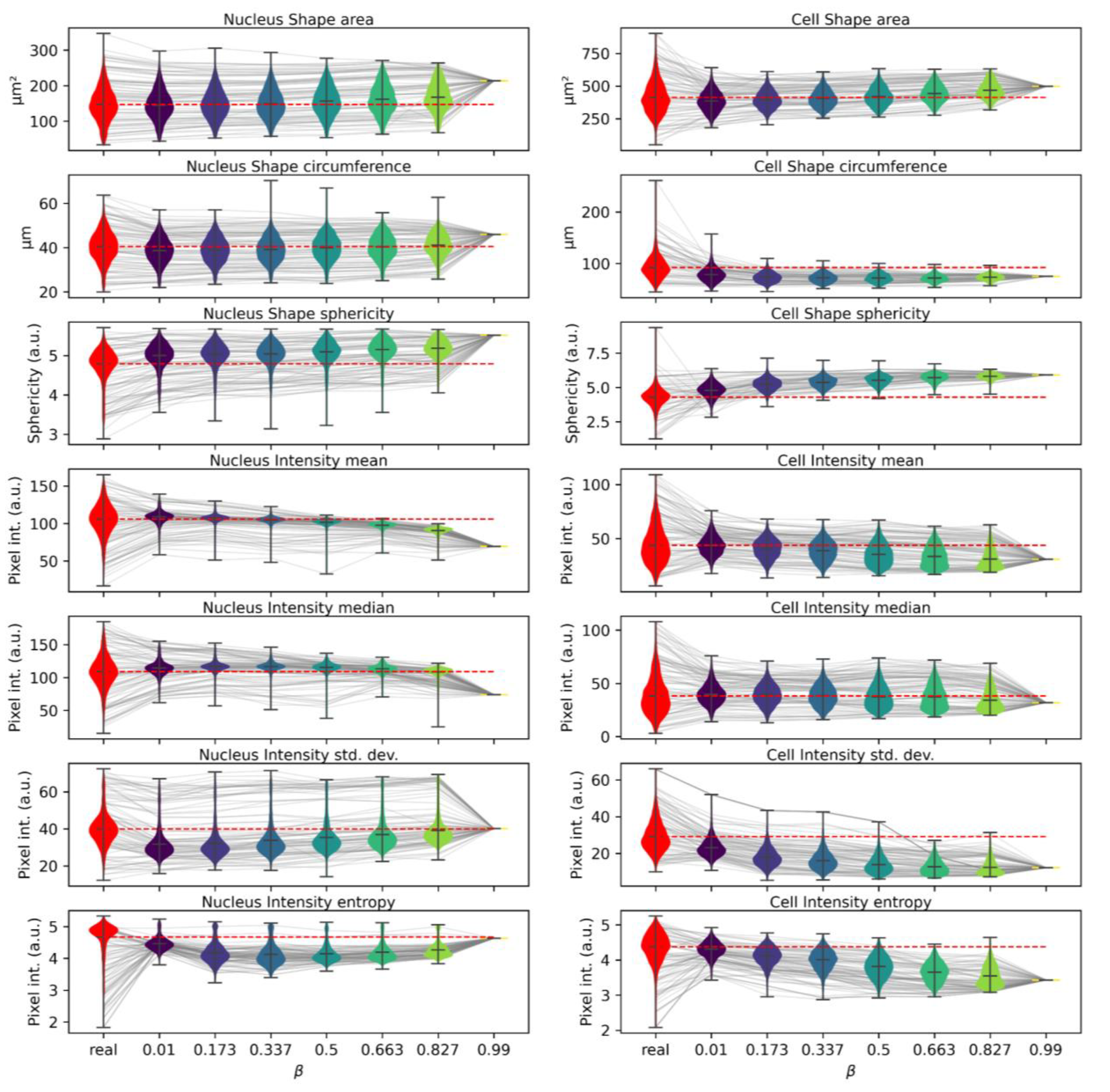

Supplement: S7 Fig — Plots on the left show seven features based on the nucleus (channel); plots on the right show the same seven features based on the cell (channel). These seven features include three shape features: area, circumference and sphericity, and four intensity-based features: median, mean, standard deviation and entropy. A grey line shows the feature value for one selected cell; connecting the feature value obtained from the real cell image with the values obtained from the generated cell images. Grey lines are plotted for a subset of all cells used in this analysis. (TIF) [file pcbi.1009155.s007.tif]

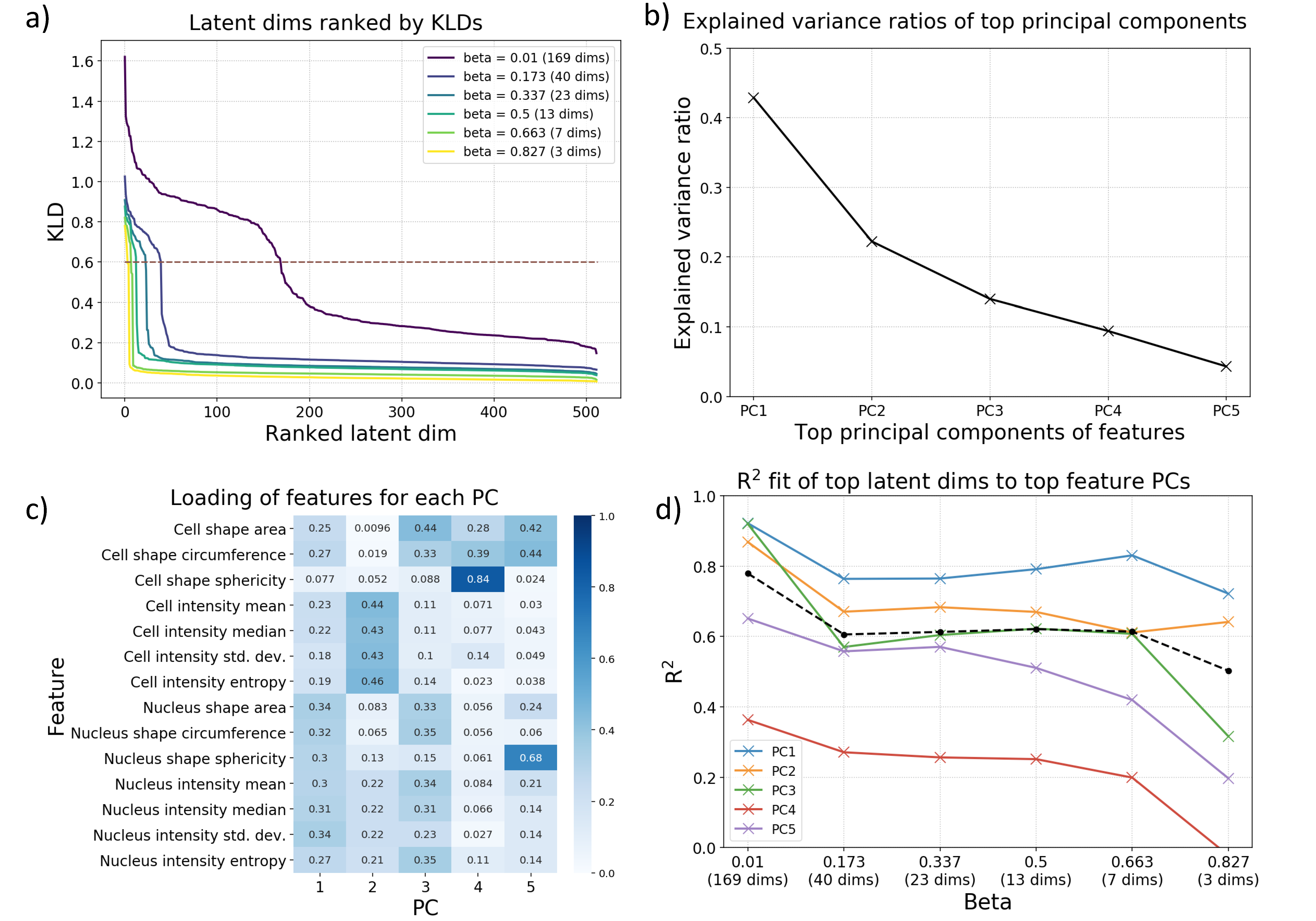

Supplement: S8 Fig — a) Ranked mean KLD per dimension for the reference latent space of the test set in selected 2D models trained with different values of β. Dimensions with a KLD larger than 0.6 are considered ‘important’ latent space dimensions. Models with low β (focus on reconstruction) have more important latent space dimensions than models with high β (focus on sparsity). b) Explained variance ratios for the top 5 principal components (PCs) of the feature space consisting of the 14 metrics derived from the real cell images for all the cells in the test set. c) Loading of each of the 14 features in the top 5 PCs of the feature space. d) R2 (explained variance) scores of linear regression models that fit the important latent space dimensions to each of the the top 5 PCs, independently, as a function of β. The black dotted line represents the total explained variance. Since the first five PCs capture 93% of the variation among the 14 features, the theoretical maximum of the black dotted line is 0.93. (TIF) [file pcbi.1009155.s008.tif]
