# Supplementary material for: A deep generative model of 3D single-cell organization
Source: PLoS Comput Biol. 2022 Jan 18;18(1):e1009155. doi: 10.1371/journal.pcbi.1009155 (PMC8797242; doi:10.1371/journal.pcbi.1009155)
Supplement: Attachment — Submitted filename: PLOS_Compbio___Statistical_Cell_CoverLetter_PointbyPoint.pdf [file pcbi.1009155.s009.pdf]

Cover letter

Dear Dr. Jeffrey J. Saucerman and Dr. Jason Haugh,

Thank you very much for overseeing the submission and revision process of our manuscript.

We thought the reviews were very constructive and have addressed all comments and requests described by the reviewers. In particular, we have added a computational experiment where we calculate various shape and intensity-based metrics from real and generated cell images. Statistical analyses, also comparing these metrics to the latent space, including new figure panels and supplementary figures, have been added to the manuscript. We discuss to what extent these biologically interpretable metrics can be captured by the generative model. Additionally, we have rewritten the results interpretation of the coupling analysis and drug perturbations sections. We place observations from these analyses in the context of established cell biology knowledge.

A point-by-point response to the reviewers' comments is found below.

We believe the valuable advice from the reviewers has helped us to substantially improve the manuscript. Please do not hesitate to let us know if we can provide any information you may need in your evaluation. We look forward to hearing from you.

With kind regards,
Theo Knijnenburg
Rory Donovan-Maiye
Greg Johnson

Point-by-point response

Reviewer #1:

Overall impressive paper that builds on previous work from this group to learn a 3D generative model from ~10^4 3D images. To my knowledge this is the largest automated analysis of 3D images yet reported. I am impressed by the scale of the dataset/analysis, the technology applied, and the biological realism of the model learned. However, at the end I am left wondering what exactly is the point of the exercise. I am not sure what can be done with this model that couldn't be done before.

Major 1.

The stated practical applications of the research are representation learning and dimensionality reduction. However, nearly the entire paper is devoted to showing the model can learn to generate cell data distributions and that the correlations between the substructures are biologically realistic. But as far as I understand it, the goal of the method is *not* to generate realistic looking cell images (see point 4), but rather to be used for feature representations and dimensionality reduction. However, as far as I can tell, the only evidence presented that these goals can be achieved with this method is in figure 6. But this section is introduced as a demonstration of the use "out of sample data" (which is commendable), which leaves the reader still wondering whether/how well does the generative model work for the original goals of representation learning and dimensionality reduction (even on the held out "in sample" data). No comparisons with other feature representation or dimensionality reduction methods are presented. Therefore, in my mind, the paper does not provide convincing evidence for the stated aims.

Response

Thank you for your feedback regarding the goals and applications of our model. As an important part of the research in our paper, we explore the two latent spaces of the trained Statistical Cell model where 3D cell images (128 x 96 x 64 = 786,432 cubic voxels) are captured with 512 latent dimensions; the reference model captures the variation in the cell and membrane channels and the conditional model captures variation in the target structures. We perform various computational experiments to interpret these latent spaces as detailed in Sections 4.2 Representing and visualization of subcellular organization via latent space embeddings, 4.3 Sparsity/Reconstruction Trade-Off, 4.4 Visualization of generated cells and conditionally generated structures and 4.5 Quantification of the coupling of subcellular structure localization to gross cellular morphology.
We have changed section titles and wording in Section 4.2, 4.3, 4.4 and 4.5 to clarify how we interpret and use the latent space representation. Moreover, we have rewritten the last paragraph of the introduction to clarify the goals and applications of our model.
Additionally, we have added a computational experiment to Section 4.3 Sparsity/Reconstruction Trade-Off to provide further insight into the interpretation of the reference latent space by correlating latent space dimensions with biological interpretable metrics as a function of beta, the parameter that controls the number of informative latent space dimensions. This analysis also addresses the reviewer's Minor point #2 and comments by other reviewers.

Major 2.

The introduction does not provide adequate context for the work.
a) What is the current state of the art for 3D images? The authors don't even distinguish between the methods applied to 3D and 2D images in the introduction.
b) what are other applications of generative models in cell image analysis? In the introduction of previous work all deep learning work is lumped together:

"learning representations of the localization of many independently labeled subcellular structures. This allows us to combine experiments of individual subcellular structures to predict distributions of fluorescent labels that are not directly observed together, creating a single model of integrated cell organization. This approach is distinct from other methods described above, as it can be used to learn and measure population distributions of cellular geometries and organelle localizations within cells, and explore their relationships to one another, as compared to prediction of an expected localization pattern in a given microscopy image."

It's not clear which methods "above" the authors are referring to. The clarity of the introduction could be improved if the authors distinguished between generative models on raw images, versus generative models built on feature spaces. Many of the limitations that the authors discuss are more specific to the latter. While the authors focus on more end-to-end and classification applications of deep learning models, there is a lot of recent work that focuses more on image generation (some of the more classic approaches e.g. Osokin et al. 2017 are cited in their discussion, but some artificial fluorescent labeling applications come to mind as well like Christiansen et al. 2018 or Ounkomoi et al. 2018). Additionally, works focus on the relationship between generative modeling and the utility of the latent space for downstream analyses (e.g. Goldsborough et al. 2017) and some specifically interrogate this in a conditional set-up, either exploiting protein-to-cell-structure relationship similar to this paper (Lu et al. 2019) or a drug-to-morphology relationship (Dai Yang et al. 2020). I'm not exactly sure what the authors mean when they say that their work complements previous deep-learning efforts - is it that their model is more statistically principled, more controllable, in a different application space (e.g. 3D images), etc? More specificity on this would be helpful.

Response

We agree with the reviewer's observations about the introduction and appreciate the opportunity to frame our work better. In line with the reviewer's suggestions, we have rewritten substantial parts of the Introduction, where we now make a distinction between images vs feature representations as a starting point for generative models.

Major 3.

While the authors show how varying the latent space of their reference structure model changes the morphology of the cell in an interpretable and constrained way, I still think it would be more useful to the reader to know how varying the latent space of the target structure affects the generated images – it seems like a major application of this work is to impute localization structures into existing images of reference cells, after all. Supporting this application, I would be interested in:
a) What kinds of variation are controlled by the latent variables in the target structure variation? Additionally, I would like to see some exploration or discussion of how this relates to the diversity of the training data. I see that the structures consist of training data from a single representative gene, so I expect that the variation will correspond to variation most commonly observed in the genes. For example, the expression level may be relatively constant within a marker versus if there were multiple markers of varying expression, so the range of intensity levels expressed by the generative model may be relatively constrained. Is this a limitation of the model? If so, it should be discussed.
b) Importantly, is the variation described by the target structure variation vector z_t shared across all targets t (e.g. would changing a variable interpreted as cell height for one structure change cell height across all structures), or is the model using factors independently depending on upon cell structure? Knowing this would help interpretation, because I would be able to know if I have to independently interpret the variables for each individual structure, or if just interpreting the variables for one structure is enough to characterize the behavior of the model across all structures.

Response

The reviewer brings up a very interesting line of research: Investigation of the latent space of the target structures, i.e. of the 19 organelles. By construction, these components (the image data of the organelles) are modeled independently of one another - there is only a (statistical) dependence with the reference structures, i.e. the nucleus and the cell. Given the enormous diversity in size, shape, location and number of copies across the 19 organelles, and given the large differences in the various imaging aspects, such as intensity distributions, across the 19 organelles, it will not generally be the case that (certain) latent dimensions encode the same (type of) variation and have a uniform interpretation across all organelles. The reviewer hints at joint modeling of organelles which would enable us to study interrelationships between organelles. This is indeed a very exciting research project which requires changes in the neural network architecture and many other aspects of the model. We are planning to use the ‘dual- edited’ cell lines in the Allen Cell collection where two organelles are fluorescently tagged for future studies towards joint modeling of organelles.
We have added text along these lines in the Discussion section.

Major 4.

A notable difference between this work and the previous work from these authors is the move away from adversarial losses (and therefore less realistic generation of images). This needs to be discussed.

Response

In the Discussion section, we explain why we moved away from adversarial losses. We have added an additional sentence for more clarification.

Minor 1.

The authors devote most of the paper to demonstrating that the model really can learn to generate 3D cell data. Further, figure 3 shows convincingly that the trade off between model complexity and realistic image generation appears as expected in their model as a function of the penalty. However, we have no baseline or expectation for how realistic these images need to be (and for what application). Hence, I would suggest that the authors reduce the emphasis on the generation of “realistic” images.

Response

We agree with the reviewer. We reduced the emphasis by changing text throughout, mostly concentrated in the Introduction.

Minor 2.

The authors investigate how their parameterization of beta induces a trade-off between the sparsity of the latent space versus the reconstruction quality of the generated images. However, one additional possibility is that the increased penalty from higher parameterizations of beta could be disincentivizing the model from learning more subtle axes of (still biologically important) variation (so while I agree that fewer features are more interpretable, there's a risk that fewer features means the model might miss important aspects of morphology.) I would be interested in seeing the correlation analysis with previous hand-crafted features presented in Fig S3A repeated over the models with different parameterizations in Section 4.3. Are there any kinds of hand-crafted features that are systematically present (e.g. correlated with) the features learned by models with lower parameterizations of beta, that aren't in models with higher parameterizations?

Response

Thank you for this excellent suggestion. We have added a novel computational analysis to compare biologically interpretable and relevant features (such as nuclear area) between the actual cell images and the

generated cell images as a function of beta. We describe and interpret this computational experiment in the revised manuscript. The reviewer is right with the intuition that models with high beta (less focus on reconstruction) show smaller correlations with the aforementioned cell features. However, this drop-off is not very steep (unless beta is very close to 1) and even models with very few important latent dimension (#dims <10, beta >0.5) can still explain a substantial amount of the variation among these cell features.

Minor 3.

The authors try to analyse what they refer to as “out of sample” data, which is commendable. However they are not clear what they mean by out of sample in this context. As far as I can tell, the new dataset was collected under the same conditions, same microscope, same markers., etc. The authors should clarify what they mean by “out of sample”. It seems like a major limitation of the model is the requirement of explicit declaration of the target type, and that the model cannot generalize to target types unseen in training data. For example, if I wanted to generate a different target structure not present in the training data, or if I wanted to generate a multi-localizing protein instead of a discrete localization, this does not seem to be possible in the author's set-up. Relating to this point, it would be very nice to have some discussion/analysis of whether the approach as implemented could be applied to different types of 3D image data from other labs, etc.

Response

In Section 4.6 Evaluation of drug perturbation effects on subcellular structures, we have added more explanation about the experimental conditions of the drug perturbation study. In the Discussion section we discuss the application of the Statistical Cell model to external data and for modeling relationships between multiple proteins.

Reviewer #2:

This is an impressive study that builds on the prior work of the Allen Institute for Cell Science. It learn improved generative models of subcellular patterns. Perhaps the most exciting aspect is the analysis of the dependence of different patterns on the cell and nuclear geometries. The authors also demonstrate that the learned model can be applied to measure changes in drug-treated cells without retraining. The manuscript would be improved by addressing more directly the question of the quality of the generated patterns.

Major 1.

A major issue in constructing generative models is how to assess how similar generated patterns are to the patterns used to train the model. While reconstruction error can give some indication of this, it does not help with evaluating new synthetic patterns. The manuscript argues that the generated patterns are “biologically plausible” but this is a very weak criterion. In the earliest work on building conditional generative models of subcellular patterns (https://doi.org/10.1002/cyto.a.20487), numerical “SLF” features that had been demonstrated to be able to distinguish all major patterns were used to determine how distinguishable the synthetic images are from the original images. These were also used as a comparison standard in the Human Protein Atlas' Project Discovery (https://doi.org/10.1038/nbt.4225). There are open source implementations of these features in matlab (https://github.com/CellProfiling/FeatureExtraction) and python (https://mahotas.readthedocs.io/en/latest/features.html). This issue is especially important since for some of the patterns used in this study (and previous work from the Allen Institute) reasonable synthetic patterns are notoriously hard to generate.

Response

We thank the reviewer for pointing out the important challenge of assessing similarity between patterns in the real cell images and generated cell images (or their latent spaces). We have added a computational experiment where we measure (using an in-house developed library) shape and intensity-based features from the real and generated cell images. These experiments were done to expand Section 4.3 Sparsity/Reconstruction Trade-Off -on 2D models- to provide further insight into the interpretation of the reference latent space by correlating latent space dimensions with these biological interpretable metrics as a function of beta, the parameter that controls the number of informative latent space dimensions. We describe these analyses in the Results and Methods section, and have added text in the Discussion section.

Major 2.

Another suggestion approach to improving evaluation is to measure the overlap between the patterns generated for different structures from the same cell/nuclear geometry. Ideally, the synthetic patterns would be somewhat distinct – patterns that are being faithfully generated should show extensive overlap among randomly generated synthetic images of the same structure and lower overlap between different structures.

Response

Thank you for this great suggestion. We hope you respect our decision to devote the analysis efforts for this revision to the analysis discussed above. We think your intuition is correct. In Figure 4, we can see three (n=3) examples of structures generated from the same cell and nuclear geometry. The nuclear envelope is ‘easy’ to predict from the nucleus (DNA stain) channel; it's predicting the envelope of a three dimensional (fluorescent) object. There is little variation between the three generated instantiations of the model and they are very similar to the real cell image. But the mitochondria, which are certainly much more uncoupled (biologically and statistically) to the nucleus and cell membrane, and more varying in overall shape, show widely different instantiations. These visual results already provide strong hints that the reviewer is correct regarding the statement about overlap between generated structure images.

We have added text along these lines in the Results section of Figure 4.

Minor 1.

The supplementary figure numbering should be adjusted so that they are numbered in the order that they are referred to in the manuscript.

Response

Yes, we will fix the figure numbering as we move closer to the publication stage.

Minor 2.

The introduction says that the work enables generation of a statistically meaningful “average” cell but this is not demonstrated in the manuscript and would be on very shaky ground since the models being learned assume independence of the patterns of the different subcellular structures and this is certainly not correct.

Response

We agree with the reviewer and have changed the wording.

Reviewer #3:

Summary:

The paper presents a method to learn a generative model of cell morphology of different subcellular structures from microscopy image data and to use such models to investigate biologically informative spatial correlations between these. Specifically, the authors first train a variant of an variational autoencoder (beta-VAE) on 3D images of cells labeled with a nuclei and plasma membrane reference marker that then i) is used to generate biologically plausible images by sampling the latent space, and ii) whose latent space is shown to correspond to interpretable cell features (such as height or cell cycle). Next, a similar VAE is trained for each of 24 subcellular markers this time conditioned on the reference marker channels, which again is shown to i) generate realistic images and ii) can be used to quantify the predictive coupling between the subcellular marker and each of its two corresponding reference channels. Finally the generalizability of the learned generative model is shown by applying it on images of a drug perturbation experiment.

Overall:

The paper is generally very well written and I enjoyed reading it.
In my opinion it is an important question of i) how to generate a data-driven statistical model of cell morphology based on microscopy images and ii) how such a data driven model can be used to actually infer biologically meaningful correlations between different substructures, and I think the authors succeeded to convincingly demonstrate that both can be achieved. The presented method based on (standard) beta-VAE is sound and the experiments that demonstrate the usefulness are well carried out and convincing. I especially liked the idea of using the generative model to quantify the "predictive coupling" of the subcellular structures and the reference channels.
Overall, I don't have any major issue with the paper and I think it is a valuable contribution to the journal - please see below my minor comments that I think should be easy to address.

Minor 1.

- In Fig2a and b, I would like to see some real example image from the training dataset for some of the depicted groups (e.g. interphase/anaphase for Fig2a and small/large cell height in Fig2b). This would be helpful in comparing with the variations generated from the latent space in Fig2c/d.

Response

We have added the suggested figures and agree with the reviewer that they are really helpful. Thank you.

Minor 2.

- In Fig2c, the feature mu_71 is meant to encode integrated DNA intensity, yet the images generated with increasing mu_71 are showing the DNA channel to get more compact (which is expected) but not to increase in in intensity (which I would have expected, due to chromatin condensation). Is this due to normalization of the shown images? Could the authors comment on this?

Response

Thank you for this sharp observation. The DNA integrated intensity feature is the total brightness of the DNA channel for a particular cell. We found that this feature correlates very strongly (but negatively) with the latent dimension (mu_71). Indeed the max project visualizations are contrast stretched. There are at least three considerations that may lead to variation in total DNA integrated intensity for our data set.

We describe these considerations in the associated results section (4.2 Representing and visualization of subcellular organization via latent space embeddings).

Minor 3.

For the perturbation experiment shown in Fig 6, I understand that the cell images in Fig 6c are images sampled from the model, correct? I think it would be helpful to additionally show at least one example images for each condition (Brefeldin and Paclitaxel) from the actual used dataset.

Response

Also here, we have added the suggested figures and agree with the reviewer that they are a useful addition to the drug perturbation results section. The cell images in Fig 6c (now Fig 6g) are real cell images; we have clarified this in the figure caption.

Minor 4.

- Was there any specific reason to choose 512 as the latent dimension? The authors write in L152 that there are different "effective latent dimensions". What is meant by "effective"? From FigS6 it appears that beyond > 128, there is only minor improvement (at least for the KL divergence).

Response

512 was chosen experimentally as a latent space size that is big enough to capture all variation. The reviewer is correct in the observation that only about 100 dimensions contain relevant information. We have added additional explanation in Section 4.2.

Minor 5.

The number of parameters of the used models should be stated in section 6.2

Response

We added the number of parameters for both the 2D and 3D models.

Minor 6.

- The overall training time for the full 3D model is (as acknowledged by the authors) quite long (2 weeks). What stopping criterion was used? Maybe the authors could add another sentence discussing this.

Response

We have added an additional sentence in the Discussion section about the stopping criterion for training.

Minor 7.

- typo in Fig2: "but not showing latent space..." -> "but now showing latent space..." ?

Response

Fixed. Thank you.
