# Supplementary material for: A deep generative model of 3D single-cell organization
Source: PLoS Comput Biol. 2022 Jan 18;18(1):e1009155. doi: 10.1371/journal.pcbi.1009155 (PMC8797242; doi:10.1371/journal.pcbi.1009155)
Supplement: Attachment — Submitted filename: PLOS_Compbio___Statistical_Cell_CoverLetter2_PointbyPoint.pdf [file pcbi.1009155.s010.pdf]

Cover letter

Dear Dr. Jeffrey J. Saucerman and Dr. Jason Haugh,

Thank you again for overseeing the submission and revision process of our manuscript.

We are happy to have successfully addressed the issues raised by Reviewers 2 and 3. We appreciate and have addressed the additional comments by Reviewer 1.

A point-by-point response to the additional comments by Reviewer 1 is found below.

We successfully uploaded our figure files to the Preflight Analysis and Conversion Engine (PACE) digital diagnostic tool. PACE indicated there were no problems with the figures. We replaced our original figures with 'PACE Corrected' figures. We have read and adhere to the additional instructions that were given below the reviewer comments.

Please do not hesitate to let us know if we can provide any information you may need in your evaluation. We look forward to hearing from you.

With kind regards,
Theo Knijnenburg
Rory Donovan-Maiye
Greg Johnson

Point-by-point response

Reviewer #1:

The authors have greatly improved the manuscript. They have clarified that the model cannot (and is not meant) to produce realistic cell images, and have now clarified how their work fits into the context of other recent work in the field. However, there are still 2 areas of the manuscript that I believe need to be improved because they give a misleading impression to the reader.

Point 1.

In the abstract, the authors claim: "Once trained, our model can be used to impute structures in cells where they were not imaged and to quantify the variation in the location of all subcellular structures..."

I believe the use of the word "impute" is very misleading here. Imputation is used in statistics to refer to the inference of actual values of variables that were not observed. Several recent studies, most famously Christiansen et al. (ref 9) have actually tried to add the labels to images that were not actually observed (could be called true imputation). However, in this study, no evidence is presented that the model can "impute" the locations of structures that were not observed. To do this, the authors would need to hold out the structure channels, and compare the locations of the observed labels to the distribution of labels in the generated images. The metric would simply be R2 or some other reconstruction accuracy. As discussed by the authors, the accuracy would be expected to be very high for the nuclear envelope (because of the tight coupling with the DNA stain) and very low for things like mitochondria and golgi. If they actually did this analysis, it would be clear that the claim in the abstract is misleading: only "tightly coupled" structures can actually be imputed, and the claim in the abstract could be qualified to appropriately reflect the number of structures that can be imputed.

If the authors do not wish to perform a reconstruction analysis and actually report the power of the model to impute the different structures, they at least have to remove or modify this claim in the abstract. Perhaps they can say that their model can predict the statistics of cellular structures that were not imaged, or predict plausible locations where structures might be. But as it stands, the claim of "imputation" is very misleading.

Response

We agree with this comment by the Reviewer. We have removed the word ‘impute’ from the Abstract and Section 4.4 ‘Visualization of generated cells and conditionally generated structures’, and have reformulated the text based on suggestions by the Reviewer.

Point 2.

The authors have now clarified that the drug treatments they present is an interesting use case of their model: can they detect the effects of the drugs on the structures the drugs are known to perturb. They show convincingly that the latent space of the model is able to detect the changes to the affected compartments.

[Note that there is no "baseline" presented for this analysis so that the reader can grasp the difficulty or potential utility of this feat. The input data for this experiment includes the labeled channels for some structures. Hence, a naive baseline approach here would be to encode maximum intensity projections of the single cell images with ImageNet features (or even classical image features) and look for changes in (say a 2D representation) of the latent space. How hard would it be to see the effects of the drug treated cells on the

expected target structure? Regardless of the baseline chosen, the reader needs some context to interpret these results. Could any latent space detect these changes?]

More importantly, the authors write in this section:

"Specifically, the microtubule latent space embeddings for the 348
paclitaxel-treated cells show a significant shift in the latent space positions of the overall 349
population, such that the centroid of the population of drug-treated cells is far removed 350
from the latent space origin (Fig. 6d)."

It is still not clear from the manuscript whether the microtubule labels were used in this analysis. Later, the authors write:

"Importantly, the results of this pilot experiment 363
suggest that the model is capable of producing reasonable latent space embeddings for 364
structures that are outside of the range of the original training set (specifically the 365
microtubules for paclitaxel-treated cells and the Golgi for brefeldin-treated cells). 366"

Does this mean that the changes in the microtubules were detected *without* microtubule labeling? If so, this is a great result, and needs to be clarified and highlighted. Although I may be confused by the wording, I don't think is what they have done. I think that the authors are referring to their experiment where they showed that the model correctly predicted no effect on the golgi for the drug that is supposed to affect microtubules. At the very least, the authors need to clarify what was done. Predicting no effect is not nearly as convincing a result as correctly predicting an effect.

I would strongly encourage the authors to hold out the microtubule data from paclitaxel-treated cells, and then see if they can predict (statistically) the effects of the drug on microtubules (using the latent space inferred from tight junctions and golgi.) This could be compared with the statistics of the microtubule staining (which they have). This type of analysis would actually demonstrate that their model can do something that ImageNet features really can't. As it stands, although I am convinced that the authors have built a beautiful model of the cell using unprecedented data, they have still not really demonstrated that this model can provide any new insight or practical utility.

Response

The previous comments by the Reviewer about the drug perturbation section were really valuable, and enabled us to substantially improve this part of our manuscript. We are thankful for the additional comments by the Reviewer as they allow us to clarify even better how this analysis was performed and how the results should be interpreted.

First of all, it is important to emphasize that we did not perform any new learning (training) for the drug perturbation analysis. We used the trained Statistical Cell model and ran the drug perturbation data through the trained model. In order to capture the cells of the drug -perturbation dataset (Fig 6a; both treated and untreated cells) in the conditional latent space (Fig 6d,e,f), i.e. represented by latent space coefficients, we need three data elements: 1) the reference channels (cell and nucleus), 2) the target channel (gfp-tagged structure), and 3) the selector variable t indicating one of the 24 organelles. This can be seen from the three arrows in Fig 1c moving into the encoder block and from there into the conditional latent space z_t.

To directly answer an important question that the Reviewer raised: “Does this mean that the changes in the microtubules were detected *without* microtubule labeling?” No, we did use the microtubule labeling. Specifically, the target channel containing the 3D images of the gfp-tagged microtubules were fed through the trained model in order to make the observation that the microtubule latent space embeddings of

paclitaxel-treated cells show a significant shift compared to untreated cells (Fig. 6d). Conversely, for tight junctions, we observed that both treated and untreated cells clustered around the origin, which indicates no substantial difference in tight junctions under the drug perturbations.

The Reviewer is right that if one computes features from the treated and untreated structure images, they will reveal differences. This is apparent from the examples in Fig. 2b, where we depicted the maximum intensity projections of the tagged structures for treated and untreated cells. In our case, we run the images directly through the Statistical Cell model to observe differences in their latent space embedding and show (by sampling real cells from the latent space) that these differences visually agree with prior biological knowledge and observations.

In Section 4.6 ‘Evaluation of drug perturbation effects on subcellular structures’ we have added a detailed explanation of how we obtained the latent space embeddings for the cells of the drug-perturbation dataset. We emphasize that image data of the gfp-tagged structure is required to place cells in the conditional latent space. These additions will substantially help the reader to understand the observations and interpretations of this analysis.
